# Antecedents of Intention to Adopt Mobile Health (mHealth) Application and Its Impact on Intention to Recommend: An Evidence from Indonesian Customers

**DOI:** 10.1155/2021/6698627

**Published:** 2021-04-30

**Authors:** Gilbert Sterling Octavius, Ferdi Antonio

**Affiliations:** ^1^Graduate School of Management, Universitas Pelita Harapan, Jakarta, Indonesia; ^2^Assistant Professor of Graduate School of Management, Universitas Pelita Harapan, Jakarta, Indonesia

## Abstract

**Introduction:**

Mobile health (mHealth) applications gain popularity due to the increasing number of mobile phone usage and internet penetration, which might help some of Indonesia's medical issues. However, the uptake of mHealth applications is still low in Indonesia. This study is aimed at understanding the factors that drive individuals to adopt mHealth applications and their impact on the intention to recommend.

**Methods:**

We applied a new model that combines three different theories with some other constructs: an extended unified theory of acceptance and use of technology, diffusion of innovation, and the internet customer trust model. The study used a cross-sectional study design with partial least squares causal modeling approach.

**Results:**

There are 787 respondents in our study, with the majority of them being female, young adults. Our model could explain 53.2% of the variance of intention to adopt while explaining 48.3% of the variance of intention to recommend. Initial trust in mHealth platform (*β* = 0.373, *p* = <0.001), facilitating conditions (*β* = 0.131, *p* = <0.01), and performance expectancy (*β* = 0.099, *p* = <0.05) are the top three most important drivers of intention to adopt mHealth applications. Lastly, importance-performance map analysis (IPMA) showed that the mHealth application's initial trust is the most important construct with a high-performance score. *Discussion*. Mobile health developers and managers need to improve initial trust in the mHealth platform, facilitating conditions, and performance expectancy when developing the applications. With a medium *Q*^2^_predict_, these factors can be applied out of the research context with medium predictive power.

## 1. Introduction

As the biggest archipelago, Indonesia has more than 13,000 islands, resulting in the unequal distribution of medical professionals on each island. World Health Organization recommends one doctor per 1000 citizens, while Indonesia has a ratio of 1 doctor per 3333 citizens. This results in Indonesia having the lowest doctors' ratio to Southeast Asia [1]. There is also a shift in Indonesia's disease patterns, where noncommunicable diseases (NCDs) are more prominent than communicable diseases. Better hygiene and access to healthcare and a more sedentary lifestyle are responsible for increasing NCDs from 37% in 1990 to 57% in 2015. It is estimated that it will cost Indonesia about $4.47 trillion (or $17,863 per capita) due to NCDs from 2012 through 2030, with cardiovascular disease accounting for 39.6% of the total loss of gross domestic product (GDP) output [2].

There are other challenges in accessing healthcare for Indonesians, such as the medical cost. The Center of National Statistics stated that in March 2019, 9.41% of Indonesians are in poverty, and families who are in poverty have 4.68 people in each house on average. Looking at the average spending of a poverty-stricken family, they spend Rp 425.250 per capita per month or equals roughly $26 per capita per month [3]. The lowest tier of national insurance costs Rp 28.000 ($1.94) per person. Therefore, each household has to dole out Rp 112.000 ($7.75) each month, or roughly 26% of their income each month for a family with four people. This causes a low uptake in national insurance applications among the poor, who need the insurance the most [4].

Other costs that plague healthcare issues in Indonesia include unaffordable hospital charges and medicine. As of 2005, the estimated cost per day for a primary hospital is around $30.36, while the estimated cost per outpatient visit is $9.25 for a primary hospital. These costs do not include drugs and diagnostic testings [5]. Lastly, 216 drugs on the National Essential Medicines List (EML) are not subjected to price controls. The median price ratio for public-sector procurement of the lowest-priced generic medications was 1.74 (74% higher than the international reference price). For most-sold generics, the median price ratio was 1.44 higher [6]. The tremendous cost of healthcare in Indonesia means that the prevention of disease is much more critical.

The rapid growth of changing technologies, especially in the digital innovation sector, has brought massive medical practice changes. Information and communication technologies (ICTs) have been implemented to assist, deliver healthcare services, or serve as a make-shift alternative for consultations during the COVID-19 pandemic [7]. Advances in technology have allowed medical practitioners and patients to benefit from a broad range of new health-related features from their mobile phones. In Indonesia, some of the essential benefits are but are not limited to the following: emergency response and disaster management, disease surveillance, support for clinicians' decisions in the place of care, and remote monitoring and patient care [8, 9]. In Indonesia, mHealth applications can promote health literacy, encouraging a more balanced lifestyle, prevention, control, and management of chronic diseases [10–12]. Besides the increasing prevalence of NCDs mentioned above, promoting health literacy is needed in Indonesia. As compared to other countries, Indonesia still has a low health literacy despite the advancement of technology [13, 14].

Mobile health applications are defined as tools that assist in medicine and public health via mobile devices. Mobile communication devices, such as cellphones, tablet computers, personal digital assistants (PDAs), and wearable devices, such as smart watches, are widely used for health care, information, and data collection [15]. One attributed reason for the rise of mobile health (mHealth) is the ubiquity of wireless technology. This is reflected by the increase in fiber optic backbone cable network length from 76,700 kilometers in 2014 to 164,769 kilometers in 2019 [16]. There are 175.4 million active internet users in Indonesia, which equals 64% internet penetration in January 2020, while mobile phone connectivity rises by 4.6% to 338.2 million users from January 2019 to January 2020. This mobile phone connectivity alone equals 124% of the total Indonesian population [17]. The “mobile leapfrogging” phenomenon is where new internet users use their mobile phones to access the internet and not personal computers. More than 78% of Indonesians use their mobile phones to access the internet, in contrast to 29% laptop or notebook users and 31% desktop computer users [18]. Increasing internet penetration and increasing mobile phone use allow more users to access mobile applications, especially mHealth applications. However, an initial survey in 2018 had shown that 67.6% out of 102 respondents had not tried any mHealth applications [19], while a more recent survey in 2020 showed that only 52% of Indonesians (out of 102 respondents) are using mHealth applications [20]. In Indonesia, there are only seven mHealth applications that are government-based, while there are 18 mHealth applications from the private sector. This number is increasing rapidly in the private sector, while this does not apply to the private sectors, indicating difficulties and challenges the governments face to expand mHealth application use [19].

Intention to use a specific technology influences technology's actual use [21]. A better understanding of factors that influence the intention to adopt mHealth applications will help developers improve and promote their use. However, such studies are scarce in Indonesia [22]. Only a few studies are made relating to health and information technology and the subsequent individual adoption models. Among that literature, the majority are focused on the healthcare professionals' use of mHealth technologies and not on the patients' perspectives [23]. While these studies are not prevalent, some studies have attempted to elucidate factors influencing patients to adopt mHealth, and the summary of several studies can be seen in Table 1.

The reason for conducting this study is a mixture of all of the reasons above. Poor healthcare access, coupled with increasing NCDs, might be alleviated by the use of ICTs. In the COVID-19 era, the utilization of ICTs is increasing, and an ever-increasing number of smartphone users helps this. However, despite all of these, the adoption of mHealth applications is still shallow. Although the government and Indonesian healthcare organizations are revamping their telemedicine policies, including mHealth applications due to the COVID-19 pandemic, this effort is still in the very early phase, with minimal progress desired. Therefore, this study is aimed at finding out why the adoption of mHealth is still low and gives suggestions to managers and mHealth developers on how to increase the use of mHealth.

A specific theoretical model needs to be developed and tested for different mHealth technologies in different user groups and settings to provide a better context-related understanding of technology adoption [21]. Analyzing the adoption of mHealth in the Indonesian population is necessary due to the low adoption number of mHealth applications despite the ubiquitous mobile phone users. This study is aimed at understanding the factors that influence the intention to adopt and its impact on the intention to recommend mHealth applications. We apply the extended unified theory of acceptance use and technology (UTAUT2), diffusion of innovation (DOI), and internet customer trust model with some modifications. Other constructs are included to propose a model to explain individuals' intention to adopt and intention to recommend mHealth applications, from the patient's point of view as well as the IT developers.

## 2. Theoretical Background

### 2.1. Unified Theory of Acceptance and Use of Technology (UTAUT2)

Venkatesh et al. [21] constructed UTAUT as a comprehensive synthesis from multiple studies about technology acceptance amongst users. Eight theories were combined to form UTAUT, which include the theory of reasoned action (TRA), technology acceptance model (TAM), motivational model (MM), the theory of planned behaviour (TPB), combined TAM and TPB (C-TAM-TPB), the model of PC utilization (MPCU), innovation diffusion theory (IDT), and social cognitive theory (SCT). This theory incorporates four primary constructs, which are performance expectancy (also known as perceived usefulness (PU) in the previous model), effort expectancy (also known as perceived ease of use (PEOU) in the previous model), social influence, and facilitating conditions. With this modified model, the obtained *R*^2^ value outperformed any individual models previously studied [21].

However, UTAUT focuses more on employee technology acceptance at the individual level and might not be suitable for mHealth applications. The model is then extended to study acceptance and use of technology in a consumer-based context [24]. Three constructs were added to the original model: hedonic motivation, price value, and habit. The constructs were moderated by age, gender, and experience only while dropping voluntariness of use as this construct does not fit into the target population studied for this model. Widely known as UTAUT2, this model performed better in explaining behavioural intention (from 56% to 74%) and technology use (from 40% to 52%) [24]. Some modifications are needed in order to incorporate UTAUT2 into this study. Consumers do not use mHealth applications for the intention of enjoyment but for particular purposes such as searching for trusted medical information, finding the best hospitals, and over the counter medicine delivery to their own houses. Tavares et al. [25] did not find an association between motivation and behavioural intention, while Bossen et al. [26] found that intrinsic motivation to use self-management tools is superior to extrinsic motivation, such as the perception of others. Therefore, in this study, we did not incorporate hedonic motivation and social influence from the constructs of UTAUT2. We decided to incorporate effort expectancy, performance expectancy, price value, facilitating condition, and intention to adopt from this construct.

### 2.2. Diffusion of Innovation (DOI)

Rogers developed this theory in 1995. It became one of the popular theories to explain the adoption of information technology (IT) and better understand how IT innovation spreads with indicators that are more accurate in indicating consumer behavior within and between communities [27]. Diffusion is defined as a process in which information about innovation flows within a community from one person to another. On the other hand, innovation is an idea, process, or technology that is considered novel to people within a particular community [28]. Although innovativeness was initially thought of as a moderator, this variable became a direct determinant of PU and PEOU [21]. Chew et al. [29] used the DOI theory to examine the use of the internet of healthcare services by family doctors, while Lee [30] conducted a qualitative study using Rogers and Singhal's theory to investigate the adoption of the computerized nursing care plan (CNCP) by nurses in Taiwan. Both studies reported that DOI is a significant predictor of mHealth technologies' adoption [29, 30]. Therefore, Rogers' innovation theory helps conceptualize technology adoption in a mHealth context. This study uses DOI as a theoretical framework to examine and explain the influence of innovation on patients' adoption of mHealth applications.

### 2.3. Social Media Brand Communication Theory

Beuker and Abbing [31] first developed this theory, which included firm-generated content (FGC) as one of the variables. In this study, the firm-generated content is understood as a form of content creations that the company fully controlled in their official webpages or social media [32]. Following the growth of the internet and mobile phone users, social media and the internet have become popular platforms for both users and customers to find helpful information about products, services, and other related information [33]. To our best knowledge, this study will be the first to incorporate FGC as a variable in assessing customers' intention to adopt mHealth applications.

### 2.4. Internet Customer Trust Model

Jarvenpaa et al. [34] pioneered the trust theory concerning behavioural intention. In the context of medical services, a patient's trust is defined as a set of beliefs regarding a doctor's behaviour based on the expectations set by former doctor's expertise, compassion, privacy, confidentiality, and reliability [35]. Trust is required between a patient and a doctor in order to achieve a mutual goal. Although some authors argue that trust is built regarding time, other researchers found a strong correlation between initial trust and behavioural intention. Customers mostly base their first intention on initial trust, especially on their first interaction [36].

In the mHealth context, initial trust consists of two main components: initial trust in the doctor and initial trust in mHealth platform. Doctors are the primary care provider, while mHealth platform is the media where online healthcare services are implemented. Initial trust in doctor is associated with the doctor's information quality and interaction quality, while initial trust in mHealth platform is associated with its services. Cao et al. [37] found that both initial trust in the doctor and initial trust in mHealth platform are significant predictors of intention to adopt mHealth. Hence, both initial trust in the doctor and initial trust in mHealth platforms are incorporated in this study.

### 2.5. Information Seeking Motive

Increased penetration of the internet has changed the way people search for their information, especially health-related issues. One survey done in Indonesia shows that mHealth users utilize their application to search for health-related information (51.06%), while only 14.05% use it to consult with health professionals [19]. Another survey also shows that 59% of patients expect mHealth to change how they seek health issues [38]. The mobile phone is a convenient measure for consumers to seek health information. Given the personal nature of ownership, mobile phones provide a safe and confidential way of seeking health information. Niederdeppe et al. [39] define information-seeking motive as consumers' active efforts to obtain specific information in response to a relevant event through the internet via website or applications. Deng et al. [40] found that health information usage is closely related to seeking behaviour intention in the mobile context. Therefore, this study includes information-seeking motive as another proposed factor that might affect the intention to adopt mHealth.

### 2.6. Perceived Technology Security

As transactions occur digitally through mHealth, information security concerns the consumers' perceptions about the platform's inability to safeguard financial or other important data about the consumers [41]. Therefore, this construct analyzes the potential feelings of uncertainty in adopting a particular technology [42]. Previous studies have shown that security concerns are barriers to adopting technologies where transaction and financial information is stored and managed [21, 42].

### 2.7. Perceived Privacy Risk

Raymond A. Bauer was the first to introduce the concept of privacy risk. He believed that every single action that one takes might cause unwanted consequences. The undesirable or unexpected aspect of this variable is that the individual cannot control this risk and harm the individual, which is called the risk due to an individual's actions [43]. Therefore, perceived risk refers to one's perception of uncertainty in the use of mHealth services and its consequences [44]. Although the perceived risk is mainly studied in business research, it is increasingly applied in healthcare sectors such as implementing electronic health records [45] and wearable medical devices [46]. In this study, only perceived privacy risk will be included as Deng et al. [40] found that perceived privacy risks and performance risk negatively correlated with the patients' trust and adoption intention towards mHealth services. As performance risk's definition is somewhat similar to performance expectancy, we decided not to include performance risk in this study (Deng et al. [40].

### 2.8. Intention to Adopt and Intention to Recommend

Intention may be described as how hard people are willing to try and how much determination they want to behave. As a result, intention to adopt refers to a person's subjective likelihood of engaging in a particular action [47], while the intention to recommend can be interpreted as a metric to measure how powerful a person desires to suggest mHealth applications to others [48]. Intention to adopt new technologies is an accurate predictor of consumer adoption rates in various contexts. They can be used to evaluate behavioral outcomes [49–51]. Measuring willingness to consider new technology can thus be viewed as a valuable way to assess the system's future performance. It is thus fundamental to our research and a key dependent variable in our conceptual framework.

Meanwhile, in the literature on mHealth applications, the intention to recommend variables has never been studied [52]. This variable has only been used to justify the adoption of mobile payments [53], electronic medical record portals [25], and fitness wearable [54]. Providers of this form of technology begin to rely on existing or future customers to spread the word about it [53]. However, because of the disproportionate focus on technology use, the intention to recommend has been overlooked by researchers [54]. We understand that word of mouth is an essential medium for disseminating information about technology. Acceptance of technology is no longer strictly a personal option. Social media sites and other online platforms have opened up new channels for the influential transmission of attitudes and even behaviors. Therefore, this research studies intend to recommend the second key dependent variable together with the intention to adopt.

We chose DOI, UTAUT2, and internet customer trust model as the main framework as DOI and UTAUT2 have been extensively studied in the adoption and recommendation of information technology, including mHealth applications [25, 48]. Internet customer trust model is chosen as the last main framework because initial trust is still a relatively new concept to be studied. Previous studies also indicate that initial trusts play a substantial and significant role in affecting mHealth applications' adoption [37]. These three models provide a robust explanatory power to adopt in their respective research [25, 37, 48]. Therefore, these three hypotheses focus on this study as the authors expect these three theories to be the major drivers of adoption and recommendation of mHealth.

### 2.9. Research Hypotheses

Innovativeness is defined as one's willingness to try every new technology [55]. Rogers and Shoemaker [56] conceptualized an individual as an innovator if they are an early adopter. Innovativeness had been proven as a significant predictor of behavioural intention to adopt new technology [57] and also as an antecedent for compatibility, performance expectancy, and effort expectancy [53]. Thus, we pose the following hypotheses:

(H1) Innovativeness positively influences the intention to adopt mHealth applications
(H2) Users with a higher level of innovativeness have a higher level of compatibility
(H3) Users with a higher level of innovativeness have a higher level of performance expectancy
(H4) Users with a higher level of innovativeness have a higher level of effort expectancy.

Compatibility measures the extent to which an innovation is deemed in line with the value of the current consumer lifestyle and current and past experiences [28]. Users will regard that technology is compatible if it is beneficial for them. It has been shown that compatibility is a direct predictor of behavioural intention to adopt new technology and reinforce performance expectancy and effort expectancy. Thus, we propose the following hypotheses:

(H5) Compatibility positively influences the intention to adopt mHealth applications
(H6) Users with a higher level of compatibility have a higher level of performance expectancy
(H7) Users with a higher level of compatibility have a higher level of effort expectancy.

Performance expectancy refers to the degree of benefits obtained by the user in adopting new technology [24]. Several studies found that performance expectancy consistently has a significant effect on behavioural intention [21, 58]. Patients tend to use mHealth because it is useful for them [59]. Thus, we pose the following hypothesis:

(H8) Performance expectancy positively influences the intention to adopt mHealth applications.

Effort expectancy refers to the degree of convenience associated with users' technology [24]. Studies have shown that effort expectancy is a predictor of intention to adopt a new technology [60, 61]. If users find mHealth technology easy to operate, they will have a higher intention to use it [24, 25, 48]. Increased operability also heightens users' expectations towards acquiring the desired performance for that technology [21]. This finding is also strengthened by other studies which elucidated that performance expectancy was predicted by effort expectancy [62, 63]. Thus, we propose the following hypothesis:

(H9) Effort expectancy positively influences performance expectancy
(H10) Effort expectancy positively influences the intention to adopt mHealth applications.

Dodds et al. [64] define price value as the trade-off perceived cognitively by consumers that consider the perceived benefits of applications and the monetary costs of using them. Empirical evidence confirms that consumers are more likely to adopt services with reasonable price values at a relatively low cost [53, 65]. Also, there is a significant relationship between price and new technology adoption [24]. Therefore, we pose the following hypothesis:

(H11) Price value positively influences the intention to adopt mHealth applications.

In this study, facilitating condition is defined as the extent to which patients or users perceive that there is an adequate technical infrastructure to support the use of network-based healthcare application services and resources that offer the necessary knowledge using network-based healthcare application services [21, 66]. Patients are more likely to consider telemedicine services to be the best way for their disease management if the system has a robust infrastructure. Several studies related to mHealth applications suggest that facilitating condition impacts intention to adopt directly [63, 67]. Thus, we propose the following hypothesis:

(H12) Facilitating conditions positively influence the intention to adopt mHealth applications.

Information-seeking motive is defined as the purposive search for information due to the need to fulfill specific goals [68]. With the fast-paced improvement in connectivity and mobile phones, they offer an alternative to getting prompt laboratory test results, medical knowledge, information about diseases, and the latest treatments. mHealth users who wish to know about their conditions or treatments are more likely to accept the technology and use it [40, 45, 69]. Thus, we propose the following hypothesis:

(H13) Information-seeking motive positively influences the intention to adopt mHealth applications.

Firm-generated content is any form of content (written, audio, visual, and combined) created by marketers on social media channels [70]. Once a consumer is aware of several markets' choices, they will choose to include that brand into the list of their choices [71]. At this stage, the marketing communication tries to positively differentiate the brand from its competitors by highlighting its attractive features and getting positive feedback from customers. As firm-generated contents have a higher perceived and more trusted source expertise, customers tend to trust their contents [72]. For users who emphasize the source of expertise, the firm-generated content will have a strong and positive impact on intention to use a particular technology. In this case, the firms studied have similar marketing and firm-generated contents, and thus, comparisons can still be made reliably (see data collection). Therefore, we propose the following hypothesis:

(H14) Firm-generated content positively influences the intention to adopt mHealth applications.

In the mHealth context, privacy risk refers to the possibility of information abuse due to mHealth services, such as information theft and leakage [73]. Personal health information, such as sex-transmitted infections, mental health issues, drug abuse, genetic information, and sexual preference, is a much more sensitive subject than other information such as age and address [74]. A potential mHealth user might not want to adopt the application if they feel that their privacy is being threatened. Thus, we propose the following hypothesis:

(H15) Perceived privacy risk negatively influences the intention to adopt mHealth applications.

Perceived security is defined as the level of trust in the internet to transmit sensitive information [41]. Security breaches are thought to prevent consumers from accessing sensitive information online significantly. Security breaches also apply to cellular lines and significantly affect cellular adoption rates [75]. Security remains the most significant concern facing internet banking adoption due to the possibility of data leakage or theft by hackers, for example. This phenomenon has been reflected in many studies where security is one of the most critical barriers faced in cellular acceptance and growth [76, 77]. If a potential mHealth user feels secure in assessing the application, there will be an increased possibility of adopting mHealth application. Therefore, we propose the following hypothesis:

(H16) Perceived security positively influences the intention to adopt mHealth applications.

Initial trust is a vital acceptance influence factor. Previous researches have examined initial trust mechanisms to reduce uncertainty in technology acceptance and the context of use [78, 79]. Research shows that users usually determine whether to adopt this service based on trust evaluation if a new service carries uncertainties or potential risk. Since much of the technology is unknown, initial trust plays an essential role in eliminating the risks and uncertainties in interactions [78]. A significant positive relationship between initial trust and use intention has been verified in previous researches [37, 78, 80]. Compared to traditional health services, health services delivered on the mobile internet involve more uncertainties and risks. So, initial trust in mHealth providers, including doctors and platforms, is essential for users to adopt a decision. Thus, we propose the following hypotheses:

(H17) Initial trust in doctor positively influences the intention to adopt mHealth applications
(H18) Initial trust in mHealth platform positively influences the intention to adopt mHealth applications.

Consumers with a greater intention to adopt new technology are more likely to become users and recommend the technology to others [53]. With sensitive topics and topics related to health, there is often a mismatch between intention and effective action [81]. Thus, it is very relevant to measure how behavioural intention to use and user behaviour can influence the intention to recommend using health-based applications. Intention to recommend is important to be included in this study because it gives an insight into the application developers on what should be improved or maintained to enhance the applications' performance. Hence, we pose the following hypothesis:

(H19) Intention to adopt positively influences the intention to recommend mHealth applications.

**Figure 1:**
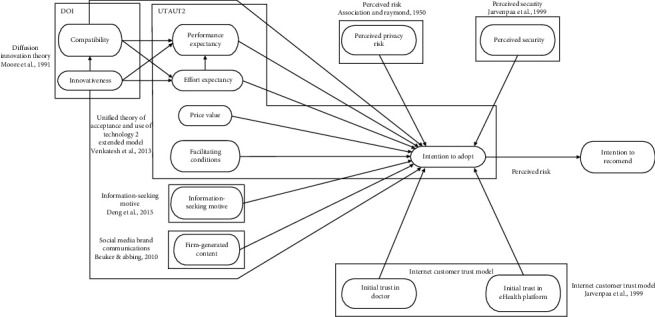
The research model. DOI: diffusion of innovation; UTAUT2: an extended unified theory of acceptance and use of technology; SMBCT: social media brand communication theory.

The 19 research hypotheses are summarized in the research model (Figure 1).

## 3. Methods

### 3.1. Survey Instrument Design

All survey items were adopted from studies regarding health information technology except for firm-generated content with some minor changes. Perceived security in this study refers to the transaction process in the mHealth application. The questionnaire items (Table 2) were first forward-translated from English to Indonesian by a certified, expert translator who is an expert in English and Indonesian languages. A panel of experts then reviewed the translated questionnaire, and some modifications took place to adjust and smoothen the translation. Another certified expert translator did the backward translation from the revised Indonesian questionnaire to English to ensure that the content did not lose its original meaning [82]. The items were measured with a 5-point Likert scale ranging from “strongly disagree” (1) to “strongly agree” (5). A preliminary test was done on ten respondents (five males and five females) who used mHealth applications before. Context-specific adjustments were then made to suit the adoption criteria of mHealth application. We performed a pilot test on 30 respondents to identify other issues and further improve the study. The data from those 40 respondents were not included in this study.

### 3.2. Data Collection

This study uses a cross-sectional study design. Our inclusion criteria include adult users' target population (above 18 years old) who had used mHealth applications at least once in the past year. This study's exclusion criteria are users who access the application via other gadgets (laptops or tablets). Due to the COVID-19 pandemic, data were collected using Google Forms from September 31 to October 15, 2020. We sent the survey link to healthcare providers, friends, and colleagues, who then shared the survey link through their contacts network (snowballing technique). At the beginning of the survey, we described the purpose of the questionnaire and explained mHealth applications' definition, and informed consents were then obtained. After that, the respondents will fill up three questionnaires that serve as the determinant for them to be included or excluded in our studies. The questions concern their age, gadgets used to access the application, and previous use of mHealth applications. If respondents are below 18 years old, access the applications via any other gadgets except mobile phones, or had never used mHealth applications before, they are excluded from the study. The questionnaires are self-filled, and one email address could only complete the form once.

There are only two Indonesian mHealth applications used in this study, and those are Halodoc© and Alodokter©. These two applications are used for the following reasons: (1) They are the two most prominent mHealth applications in Indonesia currently, and hence, comparisons can be made [83, 84]; and (2) they have similar social media activities, website performance, and several application downloads; hence, the variable user-generated content can be assessed in an apple-to-apple comparison [85]. If the respondents have used both of the applications before, they have to choose the application they access more often. We limited our sample population to residents currently staying in Jakarta, Tangerang, Bogor, Depok, and Bekasi (Jabodetabek). The reason for this demographic limitation is because these areas are well-developed in terms of infrastructure. Therefore, the results will represent the adoption of mHealth well as expanding the demographic to rural areas might bias the results towards nonadopters. Lastly, this study was not carried out in partnership with doctors or any medical professionals. We want to focus our study on the users and the application developers.

The ethics committee approved the study of the Faculty of Medicine of the University of Pelita Harapan with an ethical clearance number of 154/K-LKJ/ETIK/VIII/2020.

## 4. Results

### 4.1. Sample Characteristics

**Figure 2:**
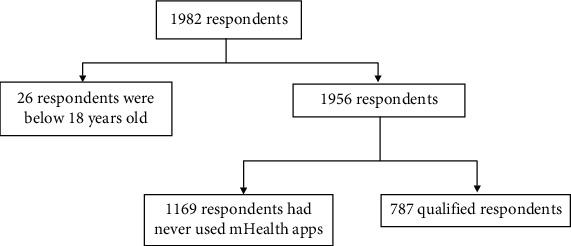
Sampling procedure and results.

**Figure 3:**
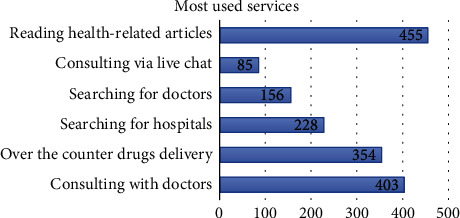
Services used by respondents. Note: Each respondent can select more than one service.

**Figure 4:**
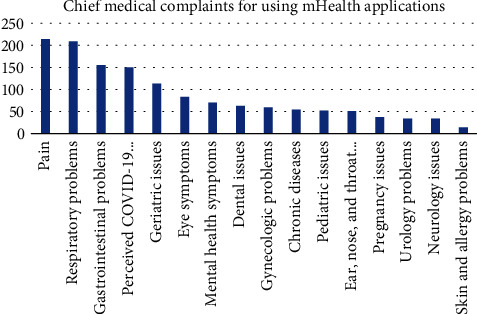
Chief medical complaints about using mHealth applications. Note: Each respondent can select more than one problem.

Figure 2 shows the sampling procedure and the results. The total number of included respondents in this study is 787 participants, and the demographic data is represented in Table 3. The majority of the participants' main characteristic is female within 18-25 years old, with monthly spending of less than Rp 3,000,000 (~$214). The mean score for intention to adopt mHealth application is high at 4.166 (SD ± 0.73). Most of the respondents (63%) use Halodoc© for their mHealth application of choice. The median score of each questionnaire, services used by the customers, and chief medical complaints about using mHealth applications are shown in Table 4 and Figures 3 and 4, respectively.

.

### 4.2. Structural Model Testing

Our model consisted of compatibility and innovativeness (DOI), performance expectancy, effort expectancy, price value, facilitating conditions (UTAUT2), information-seeking motive, firm-generated content, perceived privacy risk, perceived security, initial trust in doctor, initial trust in mHealth platform, and their effects towards intention to adopt and ultimately intention to recommend. A more detailed method of data analysis is provided in Appendices A and B.

A bootstrap with 5000 iterations of resampling was done to obtain the maximum consistency possible in the results for structural model path significance [86]. Collinearity issues were examined using the variance inflation factor (VIF), and all values are below five. Hence, collinearity is not a critical issue in this structural model [87]. The following steps will be calculating *R*^2^, path coefficients significance, and *f*^2^ effect size, which can be seen in Tables 5 and 6 [86, 87]. The blindfolding-based cross-validated redundancy measure of *Q*^2^ is assessed, predicting the data points removed for all variables [87]. With *Q*^2^ values mentioned before (see data analysis), all dependent constructs have medium relevance with the intention to adopt having the highest *Q*^2^ value (0.417).

In contrast, effort expectancy has little relevance, with a *Q*^2^ value of 0.186. Next, *f*^2^ effect size (Table 7) is assessed, and it is found that compatibility has a medium effect on effort expectancy and performance expectancy. Effort expectancy also has a medium effect on performance expectancy. Initial trust in mHealth platform has a small effect on the intention to adopt, but it is statistically significant. Innovativeness has a large effect on compatibility, and intention to adopt has a large effect on the intention to recommend.

**Figure 5:**
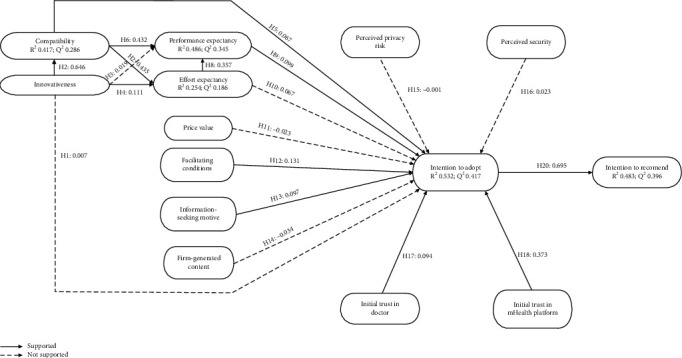
Structural model results.

Overall, compatibility, performance expectancy, facilitating conditions, information-seeking motive, initial trust in the doctor, and initial trust in mHealth platform explain 53.2% of intention to adopt mHealth. In comparison, the intention to adopt explains 48.3% of the intention to recommend mHealth applications. Initial trust in mHealth platform (*β* = 0.373, *p* = <0.001) had the strongest total effects on intention to adopt followed by facilitating conditions (*β* = 0.131, *p* = <0.01) and performance expectancy (*β* = 0.099, *p* = <0.05) (Figure 5). The firm generated content (*β* = −0.034) and price value (*β* = −0.023) had total negative effects on intention to adopt, which were not according to the predicted hypotheses, albeit they are not significant.

Shmueli et al. [88] emphasized that *R*^2^ value did not assess out-of-sample predictive power in the sense of an ability to predict the values of new cases not included in the estimation process. Therefore, Shmueli et al. [88] proposed calculating *Q*^2^_predict_ using PLSpredict with interpretation mentioned in data analysis. The results of *Q*^2^_predict_ are shown in Table 8. There are two items whose PLS-SEM RMSE values are lower than the linear regression model (LM) values; hence, this model has a medium predictive power [88].

**Figure 6:**
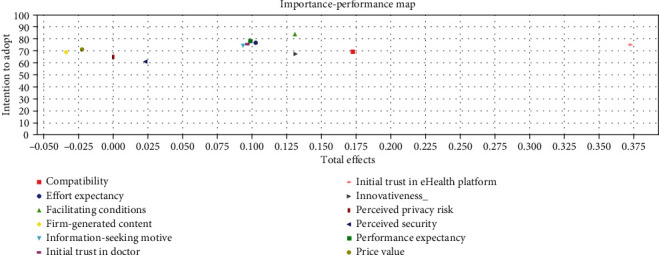
Importance-performance map (intention to adopt) for each construct.

**Figure 7:**
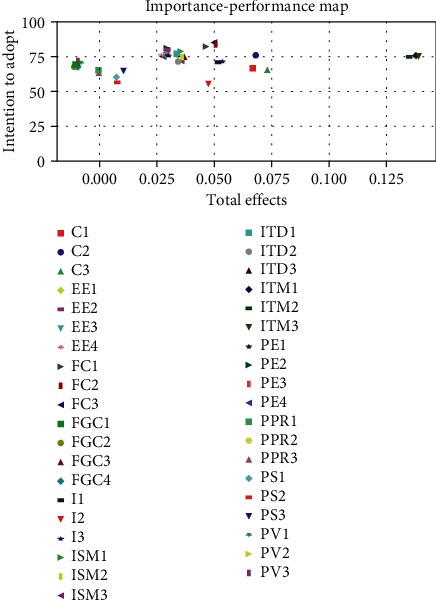
Importance-performance map (intention to adopt) for each indicator.

Lastly, importance-performance map analysis (IPMA) was done to compare the structural model's total effects on a specific target construct with its predecessors' average latent variable scores [89]. The target chosen for the IPMA is the intention to adopt, and the most important construct is initial trust in mHealth platform with an importance score of 0.373 and a performance score of 75.21 (Figure 6). This means that a one-unit increase in the performance of initial trust in the mHealth platform increases the intention to adopt the total effect's value, which is 0.373, assuming ceteris paribus. Importance-performance map analysis was also done on the indicators (Figure 7). The results showed that the initial indicator trust in mHealth platform 3 (ITM3) was the most important, with an importance score of 0.139 and a performance score of 75.03. The initial indicator trust in mHealth platform 1 (ITM1) had the best performance with a score of 75.51 and an importance score of 0.138. The second best indicator is initial trust in mHealth platform 2 (ITM2), with an importance score of 0.135 and a performance score of 75.10.

## 5. Discussion

### 5.1. Principal Findings

Compatibility, performance expectancy, facilitating conditions, information-seeking motive, initial trust in the doctor, and initial trust in the mHealth platform explained 53.2% of the variance of intention to adopt with a medium predictive power *Q*^2^_predict_. Intention to adopt explains 48.3% of the variance of intention to recommend. Our model is comparable to other studies on studying mHealth usage or adoption [37, 63]. In conclusion, the use of three major theories of UTAUT2, DOI, and internet customer trust models was successful because at least one construct had a statistically significant impact on explaining mHealth application's adoption. However, the other hypothesized theories do not impact the intention to adopt significantly. According to IPMA analysis, initial trust in mHealth platform was the most important construct and had the highest performance. Standardized coefficient-wise, our study found that initial trust in mHealth platform was the most important determinant of patients' intention to use mHealth applications, followed by facilitating conditions and performance expectancy. Other studies on mHealth applications also found that these three indicators were the primary determinant of intention to adopt [37, 63, 90].

### 5.2. Theoretical Implications

The first hypothesis (H1) is not supported as innovativeness does not significantly affect the intention to adopt. Harst et al. [91] stated in a systematic review that acceptance or rejection of innovation is a dynamic process, especially when changes in health behaviour or the adoption of mHealth applications on smartphones are involved. Thus, studies using a cross-sectional design like ours cannot capture the innovation process's role in using health care applications. This finding is also supported by Jacob et al. [92] in their systematic review, and they found that innovation does not affect mobile health tools' adoption. Regarding innovativeness on compatibility, it has a statistically significant effect and supports the second hypothesis (H2). This suggests that regular users will find it easier to incorporate mHealth applications into their own lives, which will benefit their health.

Another rejected hypothesis is the third hypothesis (H3), where innovativeness does not significantly affect performance expectancy. This finding is similar to results from earlier studies [53]. One plausible explanation was that innovativeness does not have a statistically significant effect on the intention to adopt. Hence, the indirect effect of innovativeness on adopting via performance expectancy was not statistically significant. Another hypothesis involving innovativeness is that innovativeness has a statistically significant effect on effort expectancy (H4). This finding implies that early adopters find it relatively easy to operate mHealth applications and utilize them.

All hypotheses regarding compatibility are statistically significant, from compatibility to intention to adopt (H5), compatibility to performance expectancy (H6), and compatibility to effort expectancy (H7). This finding's implication is that performance expectancy, effort expectancy, and intention to adopt are higher when the customer regards mHealth as compatible with their lives, especially in the health sector. These results are also found in previous studies [48, 53]. Effort expectancy is also found to have a statistically significant effect on performance expectancy (H8). According to Oliveira et al. [53], this means that users with lower effort in operating mHealth applications may have higher expectations of achieving gains in using mHealth-related tasks.

In our model, performance expectancy has a statistically significant effect on the intention to adopt, which suggests that users care about the advantages of using mHealth applications in their lives, supporting the ninth hypothesis (H9). Effort expectancy does not have a statistically significant effect on the intention to adopt (H10). Other studies also found this finding which evaluated medical technology adoptions [48, 63]. Tamilmani et al. [93], in a meta-analysis of UTAUT2, also cautioned about the pathway of effort expectancy to behavioural intention as the most nonsignificant path values in predicting the acceptance and use of information technology. One possible explanation for this is that most of the respondents in our study are relatively young adults (less than 35 years old), and they may be more familiar with technologies. Hence, the degree of ease of use in operating mHealth comes as more of an expectation. This was proven in one study that found effort expectancy had positive and significant effects on behavioural intention, especially among the elderly [94].

Price value does not have a statistically significant effect on the intention to adopt has a statistically significant effect on the intention to adopt, and H11 was rejected. Similar results were also found by Tavares and Oliveira [48] and Oliveira et al. [53]. The consultation fee or any other charges can be covered by using national health insurance (mandatory for all Indonesian citizens) or private insurance (54% of respondents had private insurance), which means it costs users almost nothing. Unfortunately, this fact seems to be unfamiliar to users, and hence, H11 was rejected.

In our study, facilitating conditions had a statistically significant effect on the intention to adopt, and H12 was supported. Despite the increasingly prevalent use of smartphones in Indonesia, the internet and broadband reach can still be limited in rural areas. Therefore, mHealth manufacturers should still provide customer care and assistance services to support mHealth use for users' benefit. Hypothesis 13 (H13) was supported as information-seeking motive had a statistically significant effect on the intention to adopt. This finding is supported by Alwi and Murad [69], where they provided a review on how online information seeking has affected behavioural intention. However, it is also necessary to curb inaccurate or misleading information as it might negatively affect the users' intention to adopt mHealth applications [74].

Hypothesis 14 (H14) was rejected as firm-generated content does not significantly affect the intention to adopt. One possible explanation was that consumers could assume that firm-generated content that tries to give a positive image to the company can provide an image that is too ambitious, subjective, and overwhelming for consumers. This supports previous researches that the positive tone in firm-generated content is ineffective following these findings, although these studies are not done on health technologies [95]. Perceived privacy risk did not have a statistically significant effect on the intention to adopt, and hence, H15 was rejected. The negative impact of perceived privacy risk on health information on technology acceptance intentions was inconsistent across studies. Zhang et al. [63] found that perceived privacy risk negatively affects diabetes management applications. This finding is in line with studies on mHealth services that assess acceptance behaviour [96]. A survey in America found a moderate negative effect of perceived privacy risk on patient intentions to use home health care robots [97]. However, a study in Bangladesh found that privacy did not affect the intention to adopt mHealth [62]. This finding may be attributed to the different levels of awareness of respondents' privacy protection in different regions. With the development of health information technology, patients are increasingly aware of privacy protection. Although this study found that perceived privacy risk did not significantly affect, substantial privacy protection measures were still needed [98].

In our model, perceived security does not have a statistically significant effect on the intention to adopt, and therefore, H16 was rejected. In contrast with previous research findings from Johnson et al. [99] and Oliveira et al. [53], a significant effect of perceived security on the intention to adopt was found. Some studies suggest that mobile payments are safer than traditional payment methods [100], but many consumers still consider them less secure. The gap between actual security and perceived security by users may explain the mismatch of correlation in this study [101].

Initial trust in doctor (H17) and initial trust in mHealth platform (H18) had a statistically significant effect on the intention to adopt, and they are both supported. A study shows that new service that is not commonly known and involves a large group can cause uncertainty or potential risk. Users usually decide whether to adopt this service based on trust evaluation. So, the initial trust in a health service application, including doctors and platforms, is an essential factor for users to decide whether to use such a platform [78]. Cao et al. [37] also found that their previously received medical services were primarily offline for first-time healthcare application users. The healthcare application platform has changed the medical service delivery channel, and thus, users will be paying more attention to the providers of this new underlying technology. In other words, for users who are not experienced with the platform, the risk perception comes mainly from the platform rather than the doctor. Therefore, the initial trust in the platform will have a more significant impact on adoption intentions.

Furthermore, Cao et al. [37] found that initial trust in mHealth applications had a more significant impact than initial trust in doctors, which were also found in this study. The last hypothesis (H19) was also supported as the intention to adopt had a statistically significant effect on the intention to recommend. This finding is also supported by other studies [48, 53].

### 5.3. Managerial Implications

This study considers broader and more practical antecedents that may influence mHealth application adoption and its intention to recommend mHealth application. Trust in mHealth platform is a significant adoption driver of mHealth application and the most prominent contribution amongst other constructs. This construct was also the most critical construct with a high-performance score in our IPMA analysis. One of the main reasons for not adopting mHealth in Indonesia is that consumers give low trust to mHealth applications [19]. This specific finding can provide a reference for mHealth application managers and other countries with similar problems in developing mHealth applications. From a practical perspective, these findings suggest that mHealth application managers need to show their credibility as a trusted brand. One example will be providing relevant and trusted content during the COVID-19 pandemic and articles about COVID-19 testings, signs and symptoms, and treatments. As there is much information circulating on the internet, the mHealth platform should have trustworthy information to win the customers' initial trust. This is coupled with our findings that information-seeking motive is a significant predictor of intention to adopt. Users who actively seek out information about health-related issues will be influenced by information credibility [40].

Facilitating conditions is found to be the second most crucial factor in affecting the intention to adopt mHealth. Even though there is an increasing number of smartphone users in Indonesia, internet penetration can still be shallow in rural areas [17]. Thus, application developers need to keep this in mind while trying to improve the application so that consumers with minimal bandwidth can still access the application smoothly. Managers can also set up offline or telephone-based customer centers so that users can reach out to seek help easier. Performance expectancy is also another essential factor in influencing intention to adopt mHealth applications. Application managers need to conceive this in mind when developing and promoting mHealth applications. It is relevant to emphasize the advantages the application has in helping users manage their health-related activities more efficiently. As compatibility is also a significant driver in affecting the intention to adopt, managers need to ensure that mHealth applications will fit well with the customers' lifestyles—especially in the health-related sectors.

Another essential factor to note is initial trust in doctor as it has a significant effect on the intention to adopt. Application managers need to ensure the strict criteria for doctors who can work in their company by doing a more thorough profile check, competency testing, and completeness of national-based medical doctor certifications. Another route that can be taken to increase the initial trust in doctor is to recruit senior doctors or respectable doctors in their respective fields so that patients, especially those with chronic diseases, are familiar with them. Thus, initial trust in doctors will increase.

## 6. Limitations and Future Research

First, our study was based on a web-based survey using Google Forms. We could not administer the questions directly or clarify some points when filling up the questionnaire. Our respondents were also young adults and highly educated with low to middle monthly spending; thus, prevention of diseases and healthcare awareness will be priorities. Previous studies have also shown that mHealth users are relatively young and with higher education backgrounds [19, 37, 102]. These demographic backgrounds might have influenced some of the results. Firm-generated content might not be relevant for knowledgeable or proficient users in searching for reliable sources of information. Effort expectancy might be insignificant due to the younger population being proficient in operating mHealth applications, and thus, responsiveness of mHealth applications comes as a prerequisite for them. Those with higher education might also educate themselves on privacy risks and security in mHealth applications. Second, cross-sectional studies are also not suitable for assessing or drawing a conclusion about the effect of innovativeness on the intention to adopt as innovativeness is a dynamic process [91]. Third, our model explained only 53.2% of the variance of intention to adopt and 48.3% of the variance in intention to recommend, which indicates that some other factors affecting intention to adopt and intention to recommend may have been overlooked. Future studies could include other constructs such as habit [48] and social influence [63], which positively and significantly affect behavioural intention in the respective study. Fourth, our study only looks at mHealth applications in general. It should be applied to specific mHealth applications, such as chronic disease management applications or pediatric-specific applications, with caution. Lastly, the price value impact needs to be reevaluated as some applications do not accept national and private insurance, causing the customers to pay for telemedicine services.

## 7. Conclusions

Initial trust in mHealth applications is the most critical determinant of the patient's intention to adopt mHealth applications, followed by facilitating conditions and performance expectancy. Therefore, managers and developers need to pay special attention to maintaining and increasing users' perceptions of how credible mHealth applications are. Building supporting facilities such as customer centers and increasing the application's effectiveness should also be done to promote the applications. Our study supports the use of UTAUT2, DOI, and the internet customer trust model in explaining patients' intention to adopt mHealth applications. Besides, other context-related determinants such as habit and social influence should be examined to understand better patients' intention to adopt.

## Figures and Tables

**Table 1:** Summary of studies with mHealth adoption models.

Author (year)	Theory	Dependent variable	Findings
Diño and de Guzman [110]	UTAUT and HBM	Behavioural intention for telehealth use	UTAUT constructs (especially EE) are significant influences, while gender shows no moderating effect.
Deng et al. [111]	Extended TAM, trust and perceived risk	Adoption of mHealth services	Trust, PU, and PEOU positively correlate with adoption, while privacy and performance risks negatively correlate with trust and intention to adopt.
Meng et al. [112]	Trust transfer model	mHealth service use intention	Trust in mHealth services and trust in offline health services affect intention to use positively.
Gong et al. [113]	Extended valence and trust	Adoption of OHCS	Subjective norms, trust in providers, and perceived benefit have a positive effect, while offline habits negatively affect.
Zhang et al. [63]	UTAUT	Intention to use diabetes management applications	PE and social influence are the most important determinants.
Ramírez-Correa et al. [114]	TPB and TAM	Adoption of telemedicine during COVID-19 pandemic	TPB provides a significant explanatory power.

TAM: technology acceptance model; OHCS: online health consultation service; UTAUT: unified theory of acceptance and use of technology; TPB: the theory of planned behaviour; HBM: health belief model; EE: effort expectancy; PU: perceived usefulness; PEOU: perceived ease of use.

**Table 2:** Questionnaire items of each construct.

Constructs	Items	Source
Innovativeness	I1: If I heard about new information technology, I would look for ways to experiment with it.	[57]
	I2: Among my peers, I am usually the first to try out new information technologies.	
	I3: In general, I am hesitant to try out new information technologies.	
	I4: I like to experiment with new information technologies.	

Compatibility	C1: Using mHealth application is compatible with all aspects of my lifestyle.	[115]
	C2: Using mHealth application is completely compatible with my current situation.	
	C3: I think that using mHealth application fits well with the way I like to manage my health	

Performance expectancy	PE1: mHealth application is useful to support critical aspects of my healthcare.	[68]
	PE2: mHealth application will enhance my effectiveness in managing my healthcare.	
	PE3: Using mHealth application will improve my productivity.	
	PE4: Overall, mHealth application will be useful in managing my healthcare.	

Effort expectancy	EE1: Learning how to use mHealth application is easy for me.	[48]
	EE2: My interaction with mHealth application is clear and understandable.	
	EE3: I find mHealth application easy to use.	
	EE4: It is easy for me to become skillful at using mHealth application.	

Firm generated content	FGC1: I am satisfied with the company's social media communications for mHealth applications.	[116]
	FGC2: The level of the company's social media communications for mHealth applications meets my expectations.	
	FGC3: The company's social media communications for mHealth applications are very attractive.	
	FGC4: This company's social media communications for mHealth applications perform well when compared with social media communications of other companies.	

Price value	PV1: mHealth application is reasonably priced.	[48]
	PV2: mHealth application is a good value for the money.	
	PV3: At the current price, mHealth application provides a good value.	

Facilitating conditions	FC1: I have the resources necessary to use mHealth application.	[48]
	FC2: I have the knowledge necessary to use mHealth application.	
	FC3: mHealth application is compatible with other technologies I use.	

Information seeking motive	ISM1: I have a high intention to seek health information through mHealth application.	[40]
	ISM2: I will seek health information through mHealth application in the near future.	
	ISM3: I will recommend others to seek health information through mHealth application.	

Perceived privacy risk	PPR1: It would be risky to disclose my personal health information to mHealth application.	[50]
	PPR2: There would be a high potential for loss associated with disclosing my personal health information to mHealth application.	
	PPR3: There would be too much uncertainty associated with giving my personal health information to mHealth application.	

Perceived security	PS1: I would feel secure sending sensitive information across mobile payment for mHealth application.	[53]
	PS2: Mobile payment via mHealth application is a secure means through which to send sensitive information.	
	PS3: I would feel safe providing sensitive information about myself over mHealth application via mobile payment.	

Initial trust in doctor	ITD1: I believe the doctors in the mHealth application have medical qualifications.	[37]
	ITD2: The consultation or diagnosis provided by the doctors in mHealth application is reliable.	
	ITD3: In my opinion, the doctors in the mHealth application are trustworthy.	

Initial trust in mHealth platform	ITE1: This mHealth application can fulfill its tasks.	[37]
	ITE2: This mHealth application will keep its promises.	
	ITE3: This mHealth application will keep the customers' best interests in mind.	

Intention to adopt	IA1: I intend to use mHealth application to consult health issues when needed in the future.	[37]
	IA2: I predict that I will use mHealth application to consult health issues when needed in the future.	
	IA3: I plan to use mHealth application to consult health issues when needed in the future.	

Intention to recommend	IR1: I would recommend this mHealth application to others.	([48]; [60])
	IR2: I will definitely tell others that this mHealth application is good.	
	IR3: I am willing to tell others about the good aspects of the mHealth application.	
	IR4: I will tell my friends and family about my good experiences using mHealth application.	

**Table 3:** Demographic data of the respondents (*N* = 787).

Demographic data	Frequency (%)
Sex	
Male	232 (29)
Female	555 (71)
Age (years)	
18-25	498 (63.3)
26-35	169 (21.5)
36-45	85 (10.8)
56-65	32 (4.1)
>65	3 (0.4)
Education level	
Diploma	547 (70)
Bachelor degree	151 (19)
Master's degree	69 (9)
Doctoral degree	19 (2)
Last mHealth apps usage	
<1 month ago	386 (49)
1-3 months ago	266 (34)
3-6 months ago	87 (11)
6-12 months ago	48 (6)
Monthly household spending	
<Rp 3,000,000 (~$214)	321 (41)
Rp 3,000,000–Rp 6,000,000 (~$427)	297 (38)
Rp 6,000,000–Rp 10,000,000 (~$712)	109 (14)
>Rp 10,000,000	60 (7)
Private insurance	
Yes	427 (54)
No	360 (46)
mHealth application used	
Halodoc©	494 (63)
Alodokter©	293 (37)
Increased mHealth apps use due to COVID-19	
Yes	403 (51)
No	384 (49)

**Table 4:** Descriptive results of each item in every variable studied.

Indicator	Mean	Standard deviation
I1	3.849	0.908
I2	3.216	1.179
I4	3.67	0.959
C1	3.670	0.959
C2	4.022	0.889
C3	3.602	0.998
PE1	4.018	0.866
PE2	4.255	0.798
PE3	4.197	0.823
PE4	3.995	0.943
EE1	4.028	0.857
EE2	4.202	0.788
EE3	4.278	0.727
EE4	4.278	0.717
FGC1	3.784	0.910
FGC2	3.726	0.878
FGC3	3.813	0.874
FGC4	3.694	0.874
PV1	3.841	0.840
PV2	3.831	0.797
PV3	3.879	0.797
FC1	4.304	0.715
FC2	4.337	0.706
FC3	4.400	0.679
ISM1	4.149	0.958
ISM2	4.001	1.008
ISM3	3.893	1.048
PPR1	3.623	1.051
PPR2	3.618	1.074
PPR3	3.524	1.114
PS1	3.416	1.083
PS2	3.257	1.081
PS3	3.586	0.925
ITD1	4.094	0.820
ITD2	3.846	0.896
ITD3	3.983	0.834
ITE1	4.020	0.758
ITE2	4.004	0.780
ITE3	4.001	0.791
IA1	4.166	0.733
IA2	4.136	0.753
IA3	4.133	0.774
IR1	3.961	0.851
IR2	3.980	0.854
IR3	4.079	0.806
IR4	4.051	0.808

**Table 5:** Formative indicators' quality criteria.

Construct	Item	VIF	*R* ^2^	*R* ^2^ adjusted	*Q* ^2^
Innovativeness	I1	2.253	N/A	N/A	0.345
	I2	1.605			
	I4	2.019			

Compatibility	C1	1.637	0.417	0.416	0.286
	C2	1.523			
	C3	1.634			

Performance expectancy	PE1	1.791	0.486	0.484	0.345
	PE2	2.312			
	PE3	2.669			
	PE4	1..977			

Effort expectancy	EE1	1.508	0.264	0.262	0.186
	EE2	2.961			
	EE3	3.948			
	EE4	3.149			

Firm generated content	FGC1	2.689	N/A	N/A	N/A
	FGC2	3.397			
	FGC3	3.029			
	FGC4	2.386			

Price value	PV1	3.328	N/A	N/A	N/A
	PV2	3.159			
	PV3	2.391			

Facilitating conditions	FC1	2.122	N/A	N/A	N/A
	FC2	2.327			
	FC3	2.369			

Information seeking motive	ISM1	2.442	N/A	N/A	N/A
	ISM2	2.849			
	ISM3	2.300			

Perceived privacy risk	PPR1	2.300	N/A	N/A	N/A
	PPR2	2.979			
	PPR3	2.169			

Perceived security	PS1	1.819	N/A	N/A	N/A
	PS2	2.006			
	PS3	1.974			

Initial trust in doctor	ITD1	2.132	N/A	N/A	N/A
	ITD2	2.304			
	ITD3	2.763			

Initial trust in mHealth platform	ITM1	2.778	N/A	N/A	N/A
	ITM2	2.847			
	ITM3	2.407			

Intention to adopt	IA1	2.235	0.532	0.525	0.417
	IA2	2.453			
	IA3	2.515			

Intention to recommend	IR1	3.274	0.483	0.482	0.396
	IR2	3.704			
	IR3	3.609			
	IR4	3.550			

C: compatibility; EE: effort expectancy; FC: facilitating conditions; FGC: firm-generated content; ISM: information-seeking motive; ITD: initial trust in doctor; ITM: initial trust in mHealth; I: innovativeness; IA: intention to adopt; IR: intention to recommend; PPR: perceived privacy risks; PS: perceived security; PE: performance expectancy; PV: price value; N/A: not available.

**Table 6:** Hypotheses results.

Hypothesis	Path	Beta	*t*-statistics	Results
H1	Innovativeness ➔ intention to adopt	0.007	0.169	Not supported
H2	Innovativeness ➔ compatibility	0.646	25.418^∗∗∗^	Supported
H3	Innovativeness ➔ performance expectancy	0.019	0.520	Not supported
H4	Innovativeness ➔ effort expectancy	0.111	2.873^∗∗^	Supported
H5	Compatibility ➔ intention to adopt	0.067	2.129^∗^	Supported
H6	Compatibility ➔ performance expectancy	0.432	10.502^∗∗∗^	Supported
H7	Compatibility ➔ effort expectancy	0.435	11.247^∗∗∗^	Supported
H8	Effort expectancy ➔ performance expectancy	0.357	9.697^∗∗∗^	Supported
H9	Performance expectancy ➔ intention to adopt	0.099	2.285^∗^	Supported
H10	Effort expectancy ➔ intention to adopt	0.067	1.604	Not supported
H11	Price value ➔ intention to adopt	-0.023	0.524	Not supported
H12	Facilitating conditions ➔ intention to adopt	0.131	3.109^∗∗^	Supported
H13	Information seeking motive ➔ intention to adopt	0.097	2.862^∗∗^	Supported
H14	Firm generated content ➔ intention to adopt	-0.034	0.950	Not supported
H15	Perceived privacy risk ➔ intention to adopt	-0.001	0.0032	Not supported
H16	Perceived security ➔ intention to adopt	0.023	0.726	Not supported
H17	Initial Trust in Doctor ➔ intention to adopt	0.094	2.039^∗^	Supported
H18	Initial trust in mHealth platform ➔ intention to adopt	0.373	6.856^∗∗∗^	Supported
H19	Intention to adopt ➔ intention to recommend	0.695	26.083^∗∗∗^	Supported

^∗^ *p* < 0.05; ^∗∗^*p* < 0.01; ^∗∗∗^*p* < 0.001.

**Table 7:** Values of *f*^2^ (only values above 0.02 are shown).

Path	*f* ^2^	Effect	*t*-statistics
Compatibility ➔ effort expectancy	0.1500	Medium	4.7500^∗∗^
Compatibility ➔ performance expectancy	0.1837	Medium	4.8682^∗∗^
Effort expectancy ➔ performance expectancy	0.1820	Medium	4.4027^∗∗^
Initial trust in mHealth platform ➔ intention to adopt	0.0794	Small	2.9645^∗^
Innovativeness ➔ compatibility	0.7155	Large	7.3928^∗∗^
Intention to adopt ➔ intention to recommend	0.9336	Large	6,6805^∗∗^

^∗^ *p* < 0.01; ^∗∗^*p* < 0.001.

**Table 8:** PLSpredict assessment of manifest variables.

Item	PLS-SEM		LM	PLS-SEM – LM RMSE
	RMSE	*Q* ^2^_predict_	RMSE	
IA1	0.5464	0.4462	0.5446	0.0018
IA2	0.6042	0.3581	0.6088	-0.0267
IA3	0.6193	0.3609	0.6312	-0.0119

IA: intention to adopt; RMSE: root mean square error; LM: linear model; PLS-SEM: partial least square structural equation modeling.

**Table 9:** Measurement models and factor loadings.

Construct	Item	Factor loading	*t*-value	AVE	Composite reliability	Cronbach's alpha
Innovativeness	I1	0.892	110.04^∗∗∗^	0.738	0.894	0.821
	I2	0.810	52.23^∗∗∗^			
	I4	0.872	91.31^∗∗∗^			

Compatibility	C1	0.830	55.98^∗∗∗^	0.689	0.869	0.775
	C2	0.815	51.29^∗∗∗^			
	C3	0.845	76.93^∗∗∗^			

Performance expectancy	PE1	0.815	57.94^∗∗∗^	0.717	0.910	0.868
	PE2	0.858	66.97^∗∗∗^			
	PE3	0.885	91.21^∗∗∗^			
	PE4	0.826	57.59^∗∗∗^			

Effort expectancy	EE1	0.772	43.10^∗∗∗^	0.738	0.918	0.881
	EE2	0.891	89.46^∗∗∗^			
	EE3	0.900	104.21^∗∗∗^			
	EE4	0.868	64.44^∗∗∗^			

Firm-generated content	FGC1	0.882	81.84^∗∗∗^	0.787	0.936	0.909
	FGC2	0.916	126.88^∗∗∗^			
	FGC3	0.895	94.11^∗∗∗^			
	FGC4	0.854	60.81^∗∗∗^			

Price value	PV1	0.919	113.18^∗∗∗^	0.834	0.938	0.901
	PV2	0.917	93.47^∗∗∗^			
	PV3	0.904	87.15^∗∗∗^			

Facilitating conditions	FC1	0.872	64.94^∗∗∗^	0.790	0.919	0.867
	FC2	0.896	84.64^∗∗∗^			
	FC3	0.898	96.29^∗∗∗^			

Information-seeking motive	ISM1	0.894	81.81^∗∗∗^	0.809	0.927	0.882
	ISM2	0.917	104.53^∗∗∗^			
	ISM3	0.888	78.45^∗∗∗^			

Perceived privacy risk	PPR1	0.915	15.99^∗∗∗^	0.792	0.919	0.873
	PPR2	0.921	16.85^∗∗∗^			
	PPR3	0.831	10.45^∗∗∗^			

Perceived security	PS1	0.831	36.27^∗∗∗^	0.747	0.899	0.833
	PS2	0.861	45.89^∗∗∗^			
	PS3	0.899	102.72^∗∗∗^			

Initial trust in doctor	ITD1	0.873	79.74^∗∗∗^	0.796	0.921	0.871
	ITD2	0.885	77.50^∗∗∗^			
	ITD3	0.918	122.88^∗∗∗^			

Initial trust in mHealth platform	ITM1	0.912	107.74^∗∗∗^	0.823	0.933	0.892
	ITM2	0.913	106.80^∗∗∗^			
	ITM3	0.897	117.64^∗∗∗^			

Intention to adopt	IA1	0.893	71.01^∗∗∗^	0.802	0.924	0.877
	IA2	0.894	65.50^∗∗∗^			
	IA3	0.900	70.96^∗∗∗^			

Intention to recommend	IR1	0.904	113.30^∗∗∗^	0.828	0.949	0.931
	IR2	0.917	121.36^∗∗∗^			
	IR3	0.912	93.15^∗∗∗^			
	IR4	0.908	93.62^∗∗∗^			

^∗∗∗^ *p* <0.001.

**Table 10:** Fornell-Larcker criterion: matrix of correlation constructs and the square root of AVE (in italics).

	C	EE	FC	FGC	ISM	ITD	ITE	I	IA	IR	PPR	PS	PE	PV
C	*0.830*													
EE	0.507	*0.859*												
FC	0.346	0.637	*0.889*											
FGC	0.550	0.539	0.349	*0.887*										
ISM	0.497	0.400	0.352	0.478	*0.900*									
ITD	0.456	0.449	0.406	0.485	0.431	*0.892*								
ITM	0.504	0.539	0.507	0.542	0.476	0.799	*0.907*							
I	0.646	0.392	0.238	0.468	0.475	0.338	0.374	*0.859*						
IA	0.489	0.514	0.493	0.442	0.461	0.591	0.677	0.363	*0.896*					
IR	0.553	0.506	0.426	0.516	0.518	0.589	0.672	0.483	0.695	*0.910*				
PPR	0.120	0.105	0.085	0.181	0.219	0.219	0.115	0.195	0.116	0.116	*0.890*			
PS	0.434	0.357	0.308	0.375	0.263	0.263	0.437	0.309	0.393	0.409	0.070	*0.864*		
PE	0.625	0.583	0.440	0.546	0.463	0.464	0.513	0.438	0.536	0.577	0.139	0.379	*0.847*	
PV	0.501	0.506	0.514	0.533	0.327	0.327	0.524	0.341	0.479	0.533	0.094	0.458	0.518	*0.913*

C: compatibility; EE: effort expectancy; FC: facilitating conditions; FGC: firm-generated content; ISM: information-seeking motive; ITD: initial trust in doctor; ITM: initial trust in mHealth; I: innovativeness; IA: intention to adopt; IR: intention to recommend; PPR: perceived privacy risks; PS: perceived security; PE: performance expectancy; PV: price value.

**Table 11:** Heterotrait-monotrait ratio results for discriminant validity with average HTMT computed from 5000 bootstrap samples.

	Original sample	Sample mean	Bias	5.0%^∗^	95.0%^∗^
EE ➔ C	0.606	0.606	0.000	0.551	0.661
FC ➔ C	0.423	0.424	0.001	0.360	0.481
FC ➔ EE	0.729	0.730	0.001	0.680	0.772
FGC ➔ C	0.655	0.657	0.002	0.599	0.702
FGC ➔ EE	0.595	0.593	-0.002	0.552	0.643
FGC ➔ FC	0.390	0.389	-0.001	0.327	0.440
ISM ➔ C	0.599	0.599	0.000	0.538	0.645
ISM ➔ EE	0.448	0.448	0.000	0.379	0.518
ISM ➔ FC	0.401	0.401	0.000	0.325	0.466
ISM ➔ FGC	0.534	0.533	0.000	0.489	0.586
ITD ➔ C	0.554	0.558	0.004	0.497	0.607
ITD ➔ EE	0.507	0.508	0.000	0.450	0.569
ITD ➔ FC	0.467	0.466	-0.001	0.402	0.533
ITD ➔ FGC	0.544	0.544	0.000	0.498	0.606
ITD ➔ ISM	0.492	0.493	0.001	0.430	0.548
ITM ➔ C	0.605	0.608	0.003	0.543	0.654
ITM ➔ EE	0.602	0.603	0.001	0.552	0.652
ITM ➔ FC	0.575	0.575	0.000	0.518	0.624
ITM ➔ FGC	0.600	0.601	0.001	0.548	0.649
ITM ➔ ISM	0.536	0.536	0.000	0.474	0.587
ITM ➔ ITD	0.906	0.907	0.001	0.868	0.932
I ➔ C	0.812	0.812	0.000	0.759	0.858
I ➔ EE	0.454	0.454	-0.001	0.396	0.509
I ➔ FC	0.279	0.278	0.000	0.211	0.332
I ➔ FGC	0.544	0.545	0.001	0.481	0.597
I ➔ ISM	0.559	0.559	0.000	0.504	0.614
I ➔ ITD	0.400	0.400	0.000	0.334	0.466
I ➔ ITM	0.437	0.435	-0.001	0.378	0.499
IA ➔ C	0.591	0.594	0.002	0.519	0.644
IA ➔ EE	0.579	0.579	0.000	0.522	0.629
IA ➔ FC	0.564	0.564	0.000	0.512	0.629
IA ➔ FGC	0.492	0.492	0.000	0.432	0.546
IA ➔ ISM	0.523	0.522	-0.001	0.466	0.581
IA ➔ ITD	0.674	0.674	-0.001	0.624	0.721
IA ➔ ITM	0.764	0.765	0.002	0.713	0.798
IA ➔ I	0.424	0.424	0.000	0.349	0.484
IR ➔ C	0.650	0.650	0.000	0.597	0.698
IR ➔ EE	0.552	0.551	-0.001	0.498	0.601
IR ➔ FC	0.474	0.472	-0.002	0.411	0.529
IR ➔ FGC	0.561	0.562	0.001	0.503	0.610
IR ➔ ISM	0.572	0.572	0.000	0.518	0.621
IR ➔ ITD	0.654	0.654	0.000	0.599	0.698
IR ➔ ITM	0.737	0.738	0.001	0.690	0.778
IR ➔ I	0.552	0.552	0.000	0.486	0.599
IR ➔ IA	0.768	0.768	0.000	0.715	0.809
PPR ➔ C	0.139	0.139	0.000	0.066	0.216
PPR ➔ EE	0.117	0.116	-0.001	0.055	0.175
PPR ➔ FC	0.099	0.100	0.001	0.046	0.168
PPR ➔ FGC	0.202	0.201	-0.001	0.123	0.269
PPR ➔ ISM	0.237	0.235	-0.002	0.168	0.311
PPR ➔ ITD	0.124	0.125	0.001	0.063	0.195
PPR ➔ ITM	0.134	0.135	0.001	0.068	0.204
PPR ➔ I	0.225	0.224	-0.001	0.137	0.292
PPR ➔ IA	0.127	0.127	0.000	0.069	0.202
PPR ➔ IR	0.123	0.123	0.000	0.057	0.188
PS ➔ C	0.535	0.535	0.000	0.470	0.594
PS ➔ EE	0.407	0.406	-0.001	0.349	0.464
PS ➔ FC	0.355	0.355	0.000	0.293	0.416
PS ➔ FGC	0.428	0.428	0.001	0.364	0.489
PS ➔ ISM	0.309	0.308	-0.002	0.238	0.379
PS ➔ ITD	0.504	0.505	0.001	0.438	0.558
PS ➔ ITM	0.567	0.568	0.001	0.509	0.622
PS ➔ I	0.374	0.371	-0.003	0.307	0.441
PS ➔ IA	0.449	0.450	0.001	0.381	0.501
PS ➔ IR	0.458	0.457	-0.001	0.396	0.518
PS ➔ PPR	0.079	0.086	0.007	0.034	0.155
PE ➔ C	0.758	0.760	0.002	0.705	0.799
PE ➔ EE	0.659	0.655	-0.004	0.608	0.708
PE ➔ FC	0.505	0.502	-0.003	0.456	0.584
PE ➔ FGC	0.614	0.612	-0.001	0.568	0.665
PE ➔ ISM	0.530	0.530	0.001	0.454	0.586
PE ➔ ITD	0.589	0.593	0.003	0.533	0.635
PE ➔ ITM	0.647	0.651	0.003	0.588	0.689
PE ➔ I	0.517	0.518	0.000	0.457	0.575
PE ➔ IA	0.612	0.614	0.002	0.553	0.663
PE ➔ IR	0.642	0.643	0.001	0.582	0.689
PE ➔ PPR	0.155	0.156	0.001	0.082	0.216
PE ➔ PS	0.441	0.441	0.000	0.378	0.497
PV ➔ C	0.597	0.597	0.000	0.533	0.656
PV ➔ EE	0.561	0.564	0.002	0.498	0.617
PV ➔ FC	0.581	0.581	0.001	0.523	0.631
PV ➔ FGC	0.586	0.588	0.001	0.537	0.633
PV ➔ ISM	0.364	0.364	0.000	0.301	0.430
PC ➔ ITD	0.588	0.589	0.000	0.536	0.640
PV ➔ ITM	0.688	0.688	0.000	0.636	0.725
PV ➔ I	0.394	0.392	-0.002	0.330	0.465
PV ➔ IA	0.535	0.536	0.001	0.474	0.588
PV ➔ IR	0.580	0.579	-0.001	0.522	0.625
PV ➔ PPR	0.099	0.101	0.002	0.037	0.157
PV ➔ PS	0.525	0.524	-0.001	0.463	0.581
PV ➔ PE	0.582	0.583	0.001	0.520	0.633

^∗^Neither of the confidence intervals includes the value of 1C: compatibility; EE: effort expectancy; FC: facilitating conditions; FGC: firm-generated content; ISM: information-seeking motive; ITD: initial trust in doctor; ITM: initial trust in mHealth; I: innovativeness; IA: intention to adopt; IR: intention to recommend; PPR: perceived privacy risks; PS: perceived security; PE: performance expectancy; PV: price value.

## Data Availability

The original data used to support the findings of this study are available from the corresponding author upon reasonable request.

## Appendix

### A. Data Analysis

Descriptive statistics analyzed the demographic characteristics of respondents. Testing of the research model will be carried out by SmartPLS 3.3 [103] by using partial least squares structural equation modelling (PLS-SEM). One of the main reasons for using PLS-SEM instead of covariance-based SEM (CB-SEM) is that PLS-SEM achieves greater statistical power in all sample sizes, which means that a relationship or a hypothesis is more likely to be statistically significant when it is present in the population. PLS-SEM is also used when the research is aimed at explaining and predicting a model, while CB-SEM can only be used for prediction. The analytical steps and interpretations between these two methods are similar, except that goodness-of-fit testing needs to be done in CB-SEM while not in PLS-SEM, and PLS-SEM can utilize nonnormally distributed data. In contrast, CB-SEM needs to use customarily distributed data. Lastly, CB-SEM is based on the standard factor model. In contrast, PLS-SEM is based on the composite model, is a significant difference between the two approaches, and overall is a better analytical fit for this study [86]. Before evaluating the structural model, we assessed the measurement model to evaluate construct reliability, indicator reliability, convergent validity, and discriminant validity. Acceptable reflective measurement models are defined as loadings above 0.708 [87]. Reliability was measured using composite reliability and Cronbach's alpha. Cronbach's alpha sets the lower bound by a value of 0.7, while the upper bound for internal consistency reliability is set by composite reliability with a maximum value of 0.95 [87]. Convergent validity was based on the average variance extracted (AVE) with a threshold of ≥0.50 [87]. Discriminant validity is assessed by the Fornell-Larcker criterion, where each construct's AVE should be greater than its highest correlation with any other constructs [104]. However, Henseler et al. [105] pointed out that the Fornell-Larcker criterion does not perform well and hence proposed the heterotrait-monotrait (HTMT) ratio of the correlations [105]. For conceptually similar constructs, HTMT below 0.90 is acceptable, while HTMT below 0.85 is expected for conceptually different constructs [105]. A collinearity test is done using the variance inflation factor (VIF) to assure that independent variables do not significantly correlate. Ideally, VIF values should be below 3. Variance inflation factor of ≥3-5 indicates possible collinearity issues, while VIF ≥ 5 indicates critical collinearity issues. The model's explanatory power is measured using *R*^2^, where values of 0.75, 0.5, and 0.25 are considered substantial, moderate, and weak, respectively [106]. Guidelines for measuring *f*^2^ are that values of 0.02, 0.15, and 0.35, which depict small, medium, and large *f*^2^ effect sizes, respectively [107]. Another way to assess the PLS path model's predictive accuracy is by calculating the *Q*^2^ value. As a rule of thumb, *Q*^2^ values higher than 0, 0.25, and 0.5 depict small, medium, and large predictive relevance of the PLS-path model [108]. It is recently recommended that Q^2^_predict_ is calculated because the R^2^ statistic is not represented as a measure of the model's predictive power [88]. The interpretation of *Q*^2^_predict_ is as follows: check if PLS-SEM analysis (compared to the linear regression model (LM)) yields higher prediction errors in terms of root mean squared error (RMSE) or mean absolute error (MAE) for all (no predictive power), the majority (low predictive power), the minority or the same number (medium predictive power), or none of the indicators (high predictive power) [88]. Lastly, an importance-performance map analysis (IPMA) is interpreted to compare the structural model's total effects on a specific target construct with its predecessors' average latent variable scores [89].

### B. Measurement Model Testing

The results of the measurement model can be seen in Table 9. One indicator (I3) with a factor loading below 0.708 was removed. This item was also excluded in earlier researches [57, 60]. After dropping I3, the remaining loadings are greater than 0.708, and all the items are statistically significant at <0.001. Hence, the instruments have good indicator reliability [87]. The average variance extracted (AVE) was used to assess convergent validity, and all constructs have an AVE higher than 0.50. This means the latent variable explains more than half of its indicator variance [109].  Table 10 shows the Fornell-Larcker criterion, and the results show that all indicators have good discriminant validity. Henseler et al. [105] suggest calculating the HTMT ratio of the correlations, and a threshold value of less than 0.90 was suggested for ideal HTMT. However, to avoid any ambiguity, a procedure called bootstrapping is used to arrive at a distribution of HTMT statistics and determine if it is significantly less than one [86]. If there is a value of one in the confidence interval, it indicates a lack of discriminant validity. In contrast, if the value one falls outside the interval's range, this advocates that the two constructs are empirically different [86].  Table 11 shows that our model meets this HTMT criterion.

The measurement model results indicate that the construct reliability, indicator reliability, convergent validity, and discriminant validity of the constructs are satisfactory. A structural model can be applied to this model in the next steps of evaluation.

## References

[1] Gunawan J., Aungsuroch Y. (2015). Indonesia health care system and ASEAN economic community. *International Journal of Research in Medical Sciences*.

[2] Bloom D., Chen S., McGovern M. (2015). *Economics of Non-Communicable Diseases in Indonesia*.

[3] Badan Pusat Statistik (2019). *Persentase Penduduk Miskin Maret 2019 Sebesar 9.41 Persen*.

[4] Badan Penyelenggara Jaminan Sosial Kesehatan (2021). *Tentang Kami*.

[5] World Health Organization (2005). *Estimates of unit costs for patient services for Indonesia*.

[6] Boslaugh S. (2013). *Health Care Systems around the World. A Comparative Guide*.

[7] Echeverría P., Mas Bergas M., Puig J. (2020). COVIDApp as an innovative strategy for the management and follow-up of COVID-19 cases in long-term care facilities in Catalonia: implementation study. *JMIR Public Health and Surveillance*.

[8] Davey S., Davey A., Singh J. (2014). Mobile-health approach: a critical look on its capacity to augment health system of developing countries. *Indian Journal of Community Medicine*.

[9] Déglise C., Suggs L., Odermatt P. (2012). SMS for disease control in developing countries: a systematic review of mobile health applications. *Journal of Telemedicine and Telecare*.

[10] Kreps G. (2017). The relevance of health literacy to mHealth. *Information Services & Use*.

[11] Kumar D., Arya M. (2015). mHealth is an innovative approach to address health literacy and improve patient-physician communication – an HIV testing exemplar. *Journal of Mobile Technology in Medicine*.

[12] Vaz N. (2017). Mobile health literacy to improve health outcomes in low-middle income countries. *International Journal of Reliable and Quality E-Healthcare*.

[13] Nurjanah, Mubarokah K. (2019). Health literacy and health behavior in the rural areas. *Knee Life Sciences*.

[14] Roundtable on Health Literacy (2013). *Appendix A, Health Literacy Around the World: Part 1 Health Literacy Efforts Outside of the United States*.

[15] Cipresso P., Serino S., Villani D. (2012). Is your phone so smart to affect your state? An exploratory study based on psychophysiological measures. *Neurocomputing*.

[16] Statista Research Department (2020). *Total length of fiber-optic backbone network of Telkom Indonesia 2014-2019*.

[17] Global Digital Insights (2020). *DataReportal – Global Digital Insights*.

[18] Puspitasari L., Ishii K. (2016). Digital divides and mobile internet in Indonesia: impact of smartphones. *Telematics and Informatics*.

[19] Deloitte (2019). *21^st^ Century Health Care Challenges: A Connected Health Approach*.

[20] Pusparisa Y. (2020). *Indonesia Peringkat ke-3 Global Memanfaatkan Aplikasi Kesehatan. Databoks.katadata.co.id*.

[21] Venkatesh V., Morris M. G., Davis G. B., Davis F. D. (2003). User acceptance of information technology: toward a unified view. *MIS Quarterly*.

[22] Handayani P., Meigasari D., Pinem A., Hidayanto A., Ayuningtyas D. (2018). Critical success factors for mobile health implementation in Indonesia. *Heliyon*.

[23] Angst C., Agarwal R. (2009). Adoption of electronic health records in the presence of privacy concerns: the elaboration likelihood model and individual persuasion. *MIS Quarterly*.

[24] Venkatesh V., Thong J. Y., Xu X. (2012). Consumer acceptance and use of information technology: extending the unified theory of acceptance and use of technology. *MIS Quartely*.

[25] Tavares J., Goulão A., Oliveira T. (2016). Electronic health record portals adoption: empirical model based on UTAUT2. *Informatics for Health and Social Care*.

[26] Boessen A., Vermeulen J., de Witte L. (2017). Acceptance and usability of a home-based monitoring tool of health indicators in children of people with dementia: a Proof of Principle (POP) study. *Patient Preference and Adherence*.

[27] Rogers E. M. (1995). *Diffusion of Innovations*.

[28] Rogers E. M., Singhal A., Salwen M., Stacks D. (2003). Diffusion of Innovations. *An Integrated Approach to Communication Theory dan Research*.

[29] Chew F., Grant W., Tote R. (2004). Doctors online: using diffusion of innovations theory to understand internet use. *Family Medicine*.

[30] Lee T. T. (2004). Nurses’ adoption of technology: application of Rogers’ innovation diffusion model. *Applied Nursing Research*.

[31] Beuker R., Abbing E. R. (2010). Two faces of social media: brand communication and brand research. *Design Management Review*.

[32] Kumar A., Bezawada R., Rishika R., Janakiraman R., Kannan P. K. (2016). From social to sale: the effects of firm-generated content in social media on customer behavior. *Journal of Marketing*.

[33] Li C., Bernoff J. (2011). *Groundswell: Einning in a World Transformed by Social Technologies*.

[34] Jarvenpaa S. L., Tractinsky N., Saarinen L. (1999). Consumer trust in an Internet store: a cross-cultural validation. *Journal of Computers Communications & Control*.

[35] Pearson S., Raeke L. (2000). Patients' trust in physicians: many theories, few measures, and little data. *Journal of General Internal Medicine*.

[36] McKnight H., Choudhury V., Kacmar C. (1998). The impact of initial consumer trust on intentions to transact with a web site: a trust building model. *Journal of Strategic Information Systems*.

[37] Cao Y., Zhang J., Ma L., Qin X., Li J. (2020). Examining user’s initial trust building in Mobile online health community adopting. *International Journal of Environmental Research and Public Health*.

[38] Levy D. (2014). *Emerging mHealth: paths for growth. Pwc.com*.

[39] Niederdeppe J., Hornik R., Kelly B. (2007). Examining the dimensions of cancer-related information seeking and scanning behavior. *Health Communication*.

[40] Deng Z., Liu S., Hinz O. (2015). The health information seeking and usage behavior intention of Chinese consumers through mobile phones. *Information Technology & People*.

[41] Salisbury W. D., Pearson R. A., Pearson A. W., Miller D. W. (2001). Perceived security and world wide web purchase intention. *Industrial Management & Data Systems*.

[42] Cheng T. C. E., Lam D. Y. C., Yeung A. C. L. (2006). Adoption of internet banking: an empirical study in Hong Kong. *Decision Support Systems*.

[43] Association A.R., Hancock R. S. (1960). *Dynamic Marketing for a Changing World*.

[44] Luo X., Li H., Zhang J., Shim J. (2010). Examining multi-dimensional trust and multi-faceted risk in initial acceptance of emerging technologies: an empirical study of mobile banking services. *Decision Support Systems*.

[45] Ortega Egea J., Román González M. (2011). Explaining physicians' acceptance of EHCR systems: an extension of TAM with trust and risk factors. *Computers in Human Behavior*.

[46] Yang H., Yu J., Zo H., Choi M. (2016). User acceptance of wearable devices: an extended perspective of perceived value. *Telematics and Informatics*.

[47] Fishbein, Ajzen I. (1975). *Belief, Attitude, Intention and Behaviour; an Introduction to Theory and Research*.

[48] Tavares J., Oliveira T. (2018). New integrated model approach to understand the factors that drive electronic health record portal adoption: cross-sectional national survey. *Journal of Medical Internet Research*.

[49] de Veer A., Peeters J., Brabers A., Schellevis F., Rademakers J., Francke A. (2015). Determinants of the intention to use e-Health by community dwelling older people. *BMC Health Services Research*.

[50] Karahoca A., Karahoca D., Aksöz M. (2017). Examining intention to adopt to internet of things in healthcare technology products. *Kybernetes*.

[51] Sun S., Hwang H., Dutta B., Peng M. (2019). Exploring critical factors influencing nurses’ intention to use tablet PC in patients’ care using an integrated theoretical model. *Libyan Journal of Medicine*.

[52] Or C. K., Karsh B. T. (2009). A systematic review of patient acceptance of consumer health information technology. *Journal of the American Medical Informatics Association*.

[53] Oliveira T., Thomas M., Baptista G., Campos F. (2016). Mobile payment: understanding the determinants of customer adoption and intention to recommend the technology. *Computers in Human Behavior*.

[54] Talukder M., Chiong R., Bao Y., Hayat Malik B. (2019). Acceptance and use predictors of fitness wearable technology and intention to recommend. *Industrial Management & Data Systems*.

[55] Flynn L. R., Goldsmith R. E. (1993). A validation of the Goldsmith and Hofacker innovativeness scale. *Educational and Psychological Measurement*.

[56] Rogers E., Shoemaker F. (1971). *Communication of Innovations*.

[57] Yi M. Y., Jackson J. D., Park J. S., Probst J. C. (2006). Understanding information technology acceptance by individual professionals: toward an integrative view. *Information dan Management*.

[58] Raza S. A., Umer A., Shah N. (2017). New determinants of ease of use and perceived usefulness for mobile banking adoption. *International Journal of Electronic Customer Relationship Management*.

[59] Portz J., Bayliss E., Bull S. (2019). Using the technology acceptance model to explore user experience, intent to use, and use behavior of a patient portal among older adults with multiple chronic conditions: descriptive qualitative study. *Journal of Medical Internet Research*.

[60] Miltgen C. L., Popovič A., Oliveira T. (2013). Determinants of end-user acceptance of biometrics: integrating the "Big 3" of technology acceptance with privacy context. *Decision Support Systems*.

[61] Tarhini A., El-Masri M., Ali M., Serrano A. (2016). Extending the UTAUT model to understand the customers’ acceptance and use of internet banking in Lebanon. *People*.

[62] Hoque M., Bao Y., Sorwar G. (2017). Investigating factors influencing the adoption of e-Health in developing countries: a patient’s perspective. *Informatics for Health and Social Care*.

[63] Zhang Y., Liu C., Luo S. (2019). Factors influencing patients’ intentions to use diabetes management apps based on an extended unified theory of acceptance and use of technology model: web-based survey. *Journal of Medical Internet Research*.

[64] Dodds W., Monroe K., Grewal D. (1991). Effects of price, brand, and store information on buyers’ product evaluations. *Journal of Marketing Research*.

[65] Maillet É., Mathieu L., Sicotte C. (2015). Modeling factors explaining the acceptance, actual use and satisfaction of nurses using an electronic patient record in acute care settings: an extension of the UTAUT. *International Journal of Medical Informatics*.

[66] Basoglu N., Daim T. U., Topacan U. (2010). Determining patient preferences for remote monitoring. *Journal of Medical Systems*.

[67] Hsu C. L., Tseng K. C., Chuang Y. H. (2011). Predictors of future use of telehomecare health services by middle-aged people in Taiwan. *Journal of Social Behavior and Personality*.

[68] Wilson E., Lankton N. (2004). Modeling patients’ acceptance of provider-delivered e-health. *Journal of the American Medical Informatics Association*.

[69] Alwi S., Murad M. (2018). Online information seeking: a review of the literature in the health domain. *International Journal of Computer and Information Engineering*.

[70] Stephen A. T., Galak J. (2012). The effects of traditional and social earned media on sales: a study of a microlending marketplace. *Journal of Marketing Research*.

[71] De Bruyn A., Lilien G. L. (2008). A multi-stage model of word-of-mouth influence through viral marketing. *International Journal of Research in Marketing*.

[72] Huang J., Hsiao T., Chen Y. (2012). The effects of electronic word of mouth on product judgment and choice: the moderating role of the sense of virtual community. *Journal of Applied Social Psychology*.

[73] Lim N. (2003). Consumers' perceived risk: sources versus consequences. *Electronic Commerce Research and Applications*.

[74] Bansal G., Zahedi F. M., Gefen D. (2010). The impact of personal dispositions on information sensitivity, privacy concern and trust in disclosing health information online. *Decision Support Systems*.

[75] Ghosh A. K., Swaminatha T. M. (2001). Software security and privacy risks in mobile e-commerce. *ACM*.

[76] Maduku D. K. (2016). The effect of institutional trust on internet banking acceptance: perspectives of South African banking retail customers. *South African Journal of Economic and Management Sciences*.

[77] Sun B., Sun C., Liu C., Gui C. (2017). Research on initial trust model of mobile banking users. *Journal of Risk Analysis and Crisis Response*.

[78] Gao L., Waechter K. A. (2015). Examining the role of initial trust in user adoption of mobile payment services: an empirical investigation. *Information Systems Frontiers*.

[79] Zhou T., Lu Y., Wang B. (2016). Examining online consumers’ initial trust building from an elaboration likelihood model perspective. *Information Systems Frontiers*.

[80] Kaabachi S., Mrad B. S., dan O’Leary B. (2019). Consumer’s initial trust formation in IOB’s acceptance. *The International Journal of Bank Marketing*.

[81] Tavares J., Oliveira T. (2017). Electronic Health Record Portal Adoption: a cross country analysis. *BMC Medical Informatics and Decision Making*.

[82] World Health Organization (2020). *Process of translation and adaptation of instruments*.

[83] Alodokter (2018). *Tentang Kami*.

[84] JurnalApps (2020). *Halodoc - Jurnalapps.Co.Id*.

[85] Sensor Tower (2020). *Sensor Tower - Mobile App Store Marketing Intelligence*.

[86] Hair J., Hult G. T., Ringle C., Sarstedt M. (2017). *A Primer on Partial Least Squares Structural Equation Modeling (PLS-SEM), Second edition*.

[87] Hair J., Risher J., Sarstedt M., Ringle C. (2019). When to use and how to report the results of PLS-SEM. *European Business Review*.

[88] Shmueli G., Sarstedt M., Hair J. F. (2019). Predictive model assessment in PLS-SEM: guidelines for using PLSpredict. *European Journal of Marketing*.

[89] Ringle C. M., Sarstedt M. (2016). Gain more insight from your PLS-SEM results: the importance-performance map analysis. *Industrial Management and Data Systems*.

[90] Hoque R., Sorwar G. (2017). Understanding factors influencing the adoption of mHealth by the elderly: an extension of the UTAUT model. *International Journal of Medical Informatics*.

[91] Harst L., Lantzsch H., Scheibe M. (2019). Theories predicting end-user acceptance of telemedicine use: systematic review. *Journal of Medical Internet Research*.

[92] Jacob C., Sanchez-Vazquez A., Ivory C. (2020). Social, organizational, and technological factors impacting clinicians’ adoption of mobile health tools: systematic literature review. *JMIR mHealth and uHealth*.

[93] Tamilmani K., Rana N. P., Dwivedi Y. K. (2020). Consumer acceptance and use of information technology: a meta-analytic evaluation of UTAUT2. *Information Systems Frontiers*.

[94] Cimperman M., Makovec Brenčič M., Trkman P. (2016). Analyzing older users’ home telehealth services acceptance behavior—applying an extended UTAUT model. *International Journal of Medical Informatics*.

[95] Stephen A. T., Sciandra M. R., Inman J. J. (2015). *The effects of content characteritstics on consumer engagement with branded social media content on Facebook*.

[96] Guo X., Zhang X., Sun Y. (2016). The privacy–personalization paradox in mHealth services acceptance of different age groups. *Electronic Commerce Research and Application*.

[97] Alaiad A., Zhou L. (2014). The determinants of home healthcare robots adoption: an empirical investigation. *International Journal of Medical Informatics*.

[98] Veazie S., Winchell K., Gilbert J. (2018). Rapid evidence review of mobile applications for self-management of diabetes. *Journal of General Internal Medicine*.

[99] Johnson V., Woolridge R., Wang W., Bell J. (2020). The impact of perceived privacy, accuracy and security on the adoption of mobile self-checkout systems. *Journal of Innovation Economics & Management*.

[100] Johnson V. L., Kiser A., Washington R., Torres R. (2018). Limitations to the rapid adoption of M-payment services: understanding the impact of privacy risk on M-payment services. *Computers in Human Behavior*.

[101] Hartono E., Holsapple C. W., Kim K. Y., Na K. S., Simpson J. T. (2014). Measuring perceived security in B2C electronic commerce website usage: a respecification and validation. *Decision Support Systems*.

[102] Lu C., Hu Y., Xie J. (2018). The use of mobile health applications to improve patient experience: cross-sectional study in Chinese public hospitals. *JMIR mHealth and uHealth*.

[103] Ringle C. M., Wende S., Becker J. M. (2015). *SmartPLS 3*.

[104] Fornell C. G., Larcker D. F. (1981). Evaluating structural equation models with unobservable variables and measurement error. *Journal of Marketing Research*.

[105] Henseler J., Ringle C. M., dan Sarstedt M. (2015). A new criterion for assessing discriminant validity in variance-based structural equation modeling. *Journal of the Academy of Marketing Science*.

[106] Sharma P. N., Sarstedt M., Shmueli G., Kim K. H., Thiele K. O. (2019). PLS-Based model selection: The role of alternative explanations in information systems research. *Journal of the Association for Information Systems*.

[107] Cohen J. (1988). *Statistical Power Analysis for the Behavioral Sciences*.

[108] Shmueli G., Ray S., Velasquez Estrada J. M., Shatla S. B. (2016). The elephant in the room: predictive performance of PLS models. *Journal of Business Research*.

[109] Henseler J., Ringle C. M., Sinkovics R. R. (2009). The use of partial least squares path modeling in international marketing. *Advances in International Marketing*.

[110] Diño M., de Guzman A. (2014). Using partial least squares (PLS) in predicting behavioral intention for telehealth use among Filipino elderly. *Educational Gerontology*.

[111] Deng Z., Hong Z., Ren C., Zhang W., Xiang F. (2018). What predicts patients’ adoption intention toward mHealth services in China: empirical study. *JMIR mHealth and uHealth*.

[112] Meng F., Guo X., Peng Z., Lai K., Zhao X. (2019). Investigating the adoption of mobile health services by elderly users: trust transfer model and survey study. *JMIR mHealth and uHealth*.

[113] Gong Z., Han Z., Li X., Yu C., Reinhardt J. D. (2019). Factors influencing the adoption of online health consultation services: the role of subjective norm, trust, perceived benefit, and offline habit. *Frontiers in Public Health*.

[114] Ramírez-Correa P., Ramírez-Rivas C., Alfaro-Pérez J., Melo-Mariano A. (2020). Telemedicine acceptance during the COVID-19 pandemic: an empirical example of robust consistent partial least squares path modeling. *Symmetry*.

[115] Moore G. C., Benbasat I. (1991). Development of an instrument to measure the perceptions of adopting an information technology innovation. *Information Systems Research*.

[116] Schivinski B., Dabrowski D. (2014). The effect of social media communication on consumer perceptions of brands. *Journal of Marketing Communications*.

[117] Zhang X., Yu P., Yan J., Ton A. M., Spil I. T. A. M. (2015). Using diffusion of innovation theory to understand the factors impacting patient acceptance and use of consumer e-health innovations: a case study in a primary care clinic. *BMC Health Services Research*.

[118] Kuo Y., Yen S. (2009). Towards an understanding of the behavioral intention to use 3G mobile value-added services. *Computers in Human Behavior*.

